# Human Laryngeal Infection by *Clinostomum complanatum*

**DOI:** 10.4269/ajtmh.17-0718

**Published:** 2018-01

**Authors:** Hyun Beom Song, Min-Ho Choi, Eun-Jae Chung

**Affiliations:** 1 Department of Parasitology and Tropical Medicine, Seoul National University College of Medicine, and Institute of Endemic Diseases, Seoul National University Medical Research Center, Seoul, Korea;; 2 Department of Otorhinolaryngology–Head and Neck Surgery, Seoul National University College of Medicine, Seoul, Korea

5A previously healthy 20-year-old man presented with foreign body sensation in the throat that had started 2 days before. Laryngoscopic examination revealed a motile worm on the surface of the cuneiform tubercle of the left arytenoid (Figure 1A and Supplemental video 1). Laboratory investigations revealed a white blood cell count of 5,100 cells/mm^3^ with 1% eosinophils, and all other blood cell counts, serum chemistry, electrolytes, liver function studies, and coagulation studies were normal. The worm in the larynx was removed with forceps during which the worm was partially torn. The worm was a linguiform trematode with two suckers on the ventral side (Figure 1B and Supplemental video 2). Further evaluation after fixation and staining clearly demonstrated ovary and anterior/posterior testis (Figure 1C). Based on the history of consumption of raw mullet 1 day before the symptom and morphology, the worm was identified as *Clinostomum complanatum*. The symptom was completely relieved after removal, and the patient did not report any recurrence.

**Figure 1.**
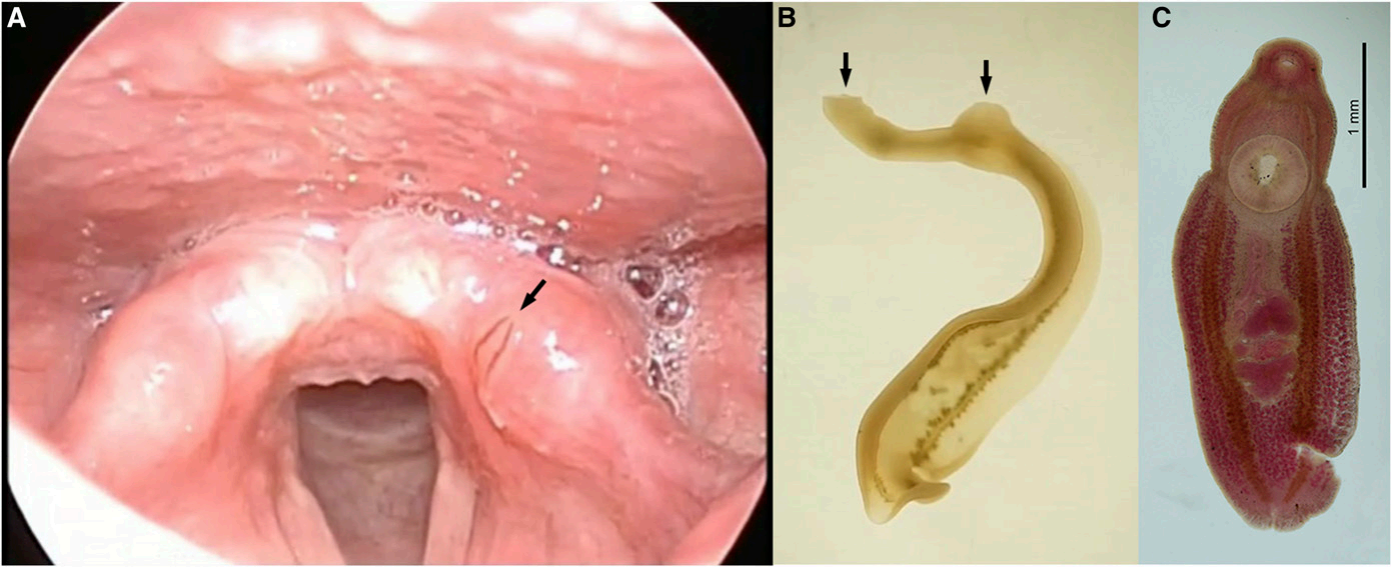
(**A**) Laryngoscopic view of a parasite on the surface of the cuneiform tubercle of the left arytenoid (black arrow). (**B**) A live worm demonstrating oral and ventral suckers during motion (black arrows). (**C**) The acetocarmine-stained *Clinostomum complanatum* demonstrating ovary and anterior/posterior testis. This figure appears in color at www.ajtmh.org.

*Clinostomum complanatum* is a digenetic trematode that resides and reproduces in the throat of definitive hosts, piscivorous birds such as herons.^1^ When the hosts thrust their beaks into the water, eggs are released into the water and hatch, and released miracidia invade the first intermediate hosts, snails. The cercariae exit snails and encyst and develop into metacercariae in the flesh of second intermediate hosts, freshwater fish. The consumption of raw freshwater fish containing metacercariae rarely infects humans where the parasites excyst in the stomach and migrate and !attach to the throat causing pharyngitis or laryngitis.^2^ Although most of the human infection cases have been reported in Korea and Japan,^2,3^ the wide distribution of freshwater fish infection has been reported in North America.^4^ Furthermore, along with a few human infection cases after consumption of raw brackish fish,^3,5^ this case suggests that brackish fish is also not completely safe to eat raw in terms of *C. complanatum* infection. A patient presenting with a globus sensation after consumption of raw fish should be appropriately evaluated including laryngoscopy.

## Supplementary Material

Supplemental Video.
